# Early cardiovascular events in women with a history of gestational diabetes mellitus

**DOI:** 10.1186/s12933-016-0338-0

**Published:** 2016-01-27

**Authors:** Karine Goueslard, Jonathan Cottenet, Anne-Sophie Mariet, Maurice Giroud, Yves Cottin, Jean-Michel Petit, Catherine Quantin

**Affiliations:** CHRU Dijon, Service de Biostatistique et d’Informatique Médicale (DIM), Université de Bourgogne, 21000 Dijon, France; Registre dijonnais des AVC, INSERM, INVS, EA4184, Univ. Bourgogne Franche-Comté, 21000 Dijon, France; Service de Cardiologie, CHRU Dijon, 21000 Dijon, France; Centre de Recherche INSERM Unité 866, Univ. Bourgogne Franche-Comté, 21000 Dijon, France; Services de diabétologie et endocrinologie, CHRU Dijon, 21000 Dijon, France; Clinical Investigation Center, clinical epidemiology/clinical trials unit, INSERM CIC 1432, Dijon University Hospital, 21000 Dijon, France; INSERM, UMR1181, Biostatistics, Biomathematics, Pharmacoepidemiology and Infectious Diseases (B2PHI), Univ. Bourgogne Franche-Comté, 21000 Dijon, France; Centre Hospitalier Universitaire, BP 77908, 21079 Dijon Cedex, France

**Keywords:** Gestational diabetes, Diabetes mellitus, Cardiovascular disease, Coronary disease

## Abstract

**Background:**

The effect of gestational diabetes mellitus (GDM) on cardiovascular diseases (CVD) is not assessed within the first 10 years postpartum, regardless of subsequent diabetes. The aim of this study was to determine the risk of CVD events related to GDM within 7 years of postpartum.

**Methods:**

This nationwide population-based study of deliveries in 2007 and 2008 with a follow-up of 7 years was based on data from the French medico-administrative database. Two groups were formed: women with a history of GDM and women without GDM or previous diabetes. CVD included angina pectoris, myocardial infarction, stroke, heart bypass surgery, coronary angioplasty, carotid endarterectomy and fibrinolysis. Hypertensive disease was assessed separately. Determinants studied included age, obesity, subsequent diabetes mellitus and hypertensive diseases during pregnancy. Adjusted odds ratios for outcomes were calculated using multiple logistic regressions.

**Results:**

The hospital database recorded 1,518,990 deliveries in 2007 and 2008. Among these, 62,958 women had a history of GDM. After adjusting for age, DM, obesity and hypertensive disorders in pregnancy, GDM was significantly associated with a higher risk of CVD (adjusted Odds Ratio aOR = 1.25 [1.09–1.43]). Considering each variable in a separate model, GDM was associated with angina pectoris (aOR = 1.68 [1.29–2.20]), myocardial infarction (aOR = 1.92 [1.36–2.71]) and hypertension (aOR = 2.72 [2.58–2.88]) but not with stroke.

**Conclusions:**

A history of GDM was identified as a risk factor of CVD, especially coronary vascular diseases, within the 7 years postpartum. A lifestyle changes from postpartum onwards can be recommended and supported.

## Background

Identifying early markers of CVD is a major challenge since CVD is responsible for 30 % of female deaths according to the WHO [1].

In France, the prevalence of the Gestational Diabetes Mellitus (GDM) ranges from 2 to 6 % [2]. GDM is defined as a high blood glucose level which appears or is diagnosed for the first time during pregnancy. Most women return to normoglycemia, but a substantial proportion of them experiences the emergence of type 2 diabetes (T2DM). The proportion reaches around 50 % within 10 years [3] and around 70 % 28 years postpartum [4, 5]. This is why GDM has been identified as a category of Diabetes Mellitus (DM) by the American Diabetes Association [6]. The principal complications of T2DM are cardiovascular diseases [7] (CVD), the risk of which is potentiated by obesity, oral contraceptives and hypertensive disorders of pregnancy [5, 8–13]. Indeed, the risk of coronary artery disease is four to six times higher in diabetic women [5].

Some studies have shown that a history of GDM increases the risk of CVD [13–15], and that the increase was mainly related to the onset of T2DM. One study found that GDM was associated with an increased risk of CVD before the onset of T2DM in women who had first-degree relatives with T2DM [16]. Evidence of the GDM effect regardless of T2DM status remains inconclusive [17, 18]. However, some studies reported that women with a history of GDM showed subclinical atherosclerosis [19], increased markers of endothelial dysfunction [20, 21], an increased risk of cardiac dysfunction [3, 7, 22, 23] and a significantly higher prevalence of metabolic syndrome very soon after pregnancy [24, 25].

These data suggest that some young women with a history of GDM may be prone to the development of cardiometabolic disorders [21] and early increases in CVD risk. These young at-risk women for CVD may benefit from preventive strategies.

For 20 years, hospital data have been used for medical research purposes and the quality of the French hospital database has been confirmed in recent studies. It provides a huge amount of epidemiological information concerning hospitalized patients in France [26–29], and can be used to create large enough cohorts to detect rare events.

The aim of the present investigation was to determine, from this hospital database, whether women with GDM have an increased risk of cardiovascular disease in the 7 years following delivery.

## Methods

### Study design

The principle of this nationwide population-based retrospective study was to examine hospital data in France for deliveries in 2007 and 2008, and CVD until 2013.

In the medico-administrative database, deliveries were identified from the codes Z37 which were considered the most reliable and extensive [30]. Two groups were formed: women who experienced GDM during pregnancy and women without GDM during pregnancy in 2007 or 2008 or in subsequent pregnancies until 2013. Women with previously diagnosed diabetes mellitus (DM) in 2007 or 2008 were excluded. If there was more than one delivery in the 2 years, hospital stays were studied one by one and taken into account if a period of at least 150 days had elapsed between two deliveries. If there was at least one case of GDM during one of the pregnancies, women were included in the GDM group. Otherwise, women were included in the group without GDM.

### Outcomes

The outcome of interest was CVD, which included angina pectoris (I20), myocardial infarction (I21-I22-I23) [28–31] but also ischemic stroke (I63–I64), transient cerebral ischemic attacks (TIA) and vascular syndromes of brain in cerebrovascular diseases (G46–G45 except G45.4) [32] coded as the main or associated diagnosis according to International Classification of Diseases (ICD-10) codes, and coronary artery bypass graft, coronary angioplasty, carotid endarterectomy (surgical removal of atheromatous plaque from an artery) and fibrinolysis (defined by the dissolution of clot with fibrinolytic agents), coded according to the French Common Classification of Procedures. When analysing ischemic stroke separately, we only considered ischemic strokes from cardio-embolism, atheroma or dissection of large arteries as carotid, vertebral or basilar arteries, microangiopathy so-called lacunar stroke induced by blood hypertension, diabetes or tobacco abuse, and undetermined ischemic (I63–I64). These cardiovascular events and therapeutic measures, coded during hospitalizations from delivery to 2013, were collected for each woman.

### Variables

The following explanatory variables were considered: maternal age, which was defined as the age of the mother at the time of birth (<20, 20–29, 30–39 and ≥40 years), hypertensive disorders of pregnancy (O10–O16), obesity ≥30 kg/m^2^ (E66) and subsequent diabetes (E10–E14) from 2007 to 2013. These last three variables were taken into account when the main or associated ICD-10 codes were found in the different hospitalizations of the same patient from the first delivery to the onset of cardiovascular event. Women aged under 12 and over 51 years were excluded from this study as the records were probably erroneous and including them would have biased the mean age at the first cardiovascular event.

### Statistical analysis

Individual characteristics are presented as proportions. Comparisons were made using a Chi squared test. The reference group included women without GDM in 2007, 2008 or until 2013. Multiple logistic regression analyses with backward selection were used to assess the association between a history of GDM and CVD.

The number of subjects required was not calculated as it was a population-based study.

SAS9.3 was used for the descriptive and multivariate analyses.

This study was approved by the National Committee for data protection (registration number 1576793). Written consent was not needed for this study. The data from the PMSI database was transmitted by the national agency for the management of hospitalization data (ATIH number 2015-111111-47-33).

## Results

The hospital database recorded 1,518,990 deliveries in 2007 and 2008 in France. Among these, 62,958 women had a history of GDM (prevalence of 4.14 %) and 3603 had previous DM.

The comparison of women’s characteristics is presented in Table 1. Women who had GDM were older (31.8 vs 29.4) and were more likely to have obesity (12.90 vs 3.87 %), subsequent DM (2.01 vs 0.12 %) and hypertensive disorders of pregnancy (7.68 vs 2.89 %). There were 229 cardiovascular events (angina pectoris, myocardial infarction, ischemic stroke, TIA and vascular syndromes of brain in cerebrovascular diseases) in the 62,958 women with a history of GDM and 3400 CVD events in the 1,452,429 women without GDM or previous DM. Angina pectoris (0.10 %), myocardial infarction (0.04 %) and ischemic stroke including TIA (0.22 %) were significantly more frequent in the GDM group. The frequency of hypertension was also significantly higher among women with a history of GDM. On average, women with a history of GDM were 39.2 years old [Standard Deviation (SD) 6.6] at the first episode of angina pectoris, 40.6 years old (SD 6.0) at the first myocardial infarction, and 36.3 years old (SD 5.6) at the first ischemic stroke or TIA. The average time between delivery and the first cardiovascular event was 3.4 years (SD 1.8) for angina pectoris, 3.8 years (SD 1.8) for myocardial infarction and 3.2 (SD 1.9) for ischemic stroke and TIA.

**Table 1:** Characteristics and adverse outcomes in women with history of GDM

	GDM 2007–2008		No GDM 2007–2008		p value
	(n = 62,958)		(n = 1,452,429)		
	Size	(%)	Size	(%)	
Age					<0.0001
<20	520	0.83	42,313	2.91	
20–29	21,471	34.10	706,688	48.66	
30–39	35,922	57.06	656,693	45.21	
≥40	5045	8.01	46,735	3.22	
Obesity	8122	12.90	56,142	3.87	<0.0001
Subsequent diabetes	1266	2.01	1674	0.12	<0.0001
Hypertensive disorders during pregnancy	4834	7.68	42,012	2.89	<0.0001
Angina pectoris	64	0.10	570	0.04	<0.0001
Myocardial infarction					
Acute myocardial infarction	26	0.04	257	0.02	<0.0001
Repeated myocardial infarction	0	0	2	0	1
Complications of acute myocardial infarction	0	0	11	0	1
Stroke					
Ischemic stroke	71	0.11	1181	0.09	<0.05
Vascular syndromes of brain in cerebrovascular diseases	9	0.01	160	0.01	0.34
Transient cerebral ischemic attacks	59	0.09	1219	0.08	0.06
Hypertensives diseases	1566	2.49	10,586	0.73	<.0001
Therapeutic measures^a^	32	0.05	236	0.02	<.0001

*GDM* gestational diabetes mellitus

^a^Coronary artery bypass graft, coronary angioplasty, carotid endarterectomy and fibrinolysis

**Fig. 1:**
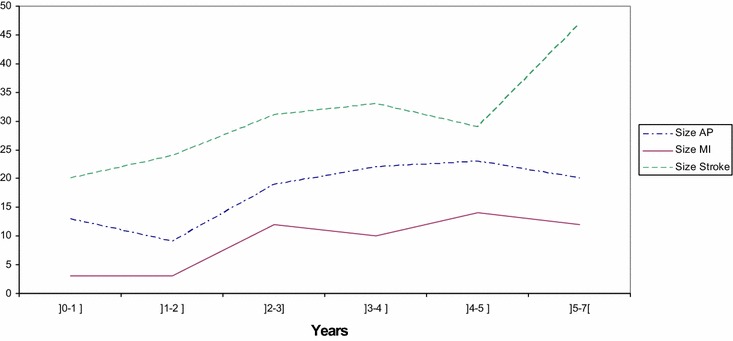
Number of cases by interval time after delivery for women with a history of GDM. *AP* angina pectoris, *MI* myocardial infarction, *stroke* ischemic stroke, TIA and vascular syndromes of brain in cerebrovascular diseases

The number of cases of angina pectoris, myocardial infarction or ischemic stroke and TIA according to time after delivery is presented in Fig. 1 for women who experienced GDM.

The results of the logistic regression analysis adjusted for age showed that GDM was significantly associated with a higher risk of CVD (Table 2). Considering each variable in a separate model, GDM significantly increased the risk of angina pectoris (adjusted Odds Ratio aOR = 2.05, 95 % Confidence Interval [1.58–2.67]), myocardial infarction (aOR = 2.07 [1.47–2.90]), and hypertensive diseases (aOR = 2.92 [2.77-3.08]) but not the risk of stroke (defined by ischemic strokes, TIA and vascular syndromes of the brain). When analyzing only ischemic strokes, GDM significantly increased the risk of these diseases (aOR = 1.28 [1.01–1.62]). Table 3 presents the results of the logistic regression analysis adjusted not only for age but also for obesity, subsequent diabetes and hypertensive disorders of pregnancy. CVD overall (aOR = 1.25 [1.09–1.43]) as well as angina pectoris (aOR = 1.68 [1.29–2.20]), myocardial infarction (aOR = 1.92 [1.36–2.71]) and hypertensive diseases (aOR = 2.72 [2.58–2.88]) were still significantly associated with GDM.

**Table 2:** Association between gestational diabetes mellitus and cardiovascular diseases adjusted for age—Multivariate logistic regressions

	Model 1: CVD	Model 2: angina pectoris	Model 3: myocardial infarction	Model 4: ischemic stroke and TIA	Model 5: ischemic stroke	Model 6: hypertensive diseases
	aOR [95 % CI]	aOR [95 % CI]	aOR [95 % CI]	aOR [95 % CI]		aOR [95 % CI]
Gestational diabetes mellitus age (ref = 21–29)	1.39 [1.21–1.59]	2.05 [1.58–2.67]	2.07 [1.47–2.90]	–	1.28 [1.01–1.62]	2.92 [2.77–3.08]
<20	0.48 [0.35–0.67]	0.29 [0.09–0.91]	0.71 [0.22–2.26]	0.50 [0.35–0.72]	0.73 [0.47–1.13]	0.44 [0.36–0.54]
30–39	1.54 [1.43–1.65]	2.06 [1.72–2.47]	3.32 [2.55–4.32]	1.35 [1.25–1.47]	1.47 [1.31–1.65]	1.95 [1.87–2.03]
≥40	2.99 [2.63–3.40]	7.10 [5.53–9.12]	9.55 [6.69–13.64]	1.99 [1.68–2.37]	2.23 [1.76–2.83]	4.63 [4.34–4.93]

*CVD* cardiovascular diseases (including angina pectoris, myocardial infarction, stroke, heart bypass, coronary angioplasty, carotid and endarterectomy and fibrinolysis intervention), *aOR* adjusted odds ratio, *CI* confidence interval

**Table 3:** Association between gestational diabetes mellitus and cardiovascular diseases adjusted for age, obesity, subsequent diabetes and hypertensive diseases during pregnancy—Multivariate logistic regressions

	Model 1: CVD	Model 2: angina pectoris	Model 3: myocardial infarction	Model 4: ischemic stroke and TIA	Model 5: ischemic stroke	Model 6: hypertensive diseases
	aOR [95 % CI]	aOR [95 % CI]	aOR [95 % CI]	aOR [95 % CI]		aOR [95 % CI]
Gestational diabetes mellitus age (ref = 21–29)	1.25 [1.09–1.43]	1.68 [1.29–2.20]	1.92 [1.36–2.71]	–	–	2.72 [2.58–2.88]
<20	0.49 [0.35–0.68]	0.29 [0.09–0.92]	0.71 [0.22–2.25]	0.50 [0.35–0.72]	0.73 [0.47–1.14]	0.44 [0.35–0.54]
30–39	1.54 [1.44–1.66]	2.07 [1.73–2.48]	3.32 [2.55–4.32]	1.36 [1.25–1.47]	1.48 [1.32–1.66]	1.95 [1.87–2.03]
≥40	2.93 [2.57–3.34]	6.98 [5.43–8.96]	9.52 [6.67–13.60]	1.96 [1.65–2.33]	2.23 [1.76–2.83]	4.63 [4.34–4.93]
Obesity	1.53 [1.34–1.75]	2.18 [1.67–2.85]	–	1.34 [1.13–1.58]	1.34 [1.06–1.69]	–
Subsequent diabetes	2.44 [1.66–3.58]	3.57 [1.91–6.70]	5.45 [2.38–12.45]	–	–	5.61 [4.85–6.50]
Hypertensive disorders during pregnancy	1.78 [1.55–2.05]	1.87 [1.39–2.55]	–	1.63 [1.37–1.95]	1.72 [1.35–2.20]	–

*CVD* cardiovascular diseases (including angina pectoris, myocardial infarction, stroke, heart bypass, coronary angioplasty, carotid and endarterectomy and fibrinolysis intervention), *aOR* adjusted odds ratio, *CI* confidence interval

## Discussion

The novelty and interest of the study lies in the fact that it shows the association between a history of GDM and cardiovascular events during the 7 years after delivery, especially myocardial infarction and angina pectoris, independently of the occurrence of DM.

We acknowledge that the present study has several limitations. First, the study focused only on women who had been hospitalized between 2007 and 2013 and for CVD, including angina pectoris, which does not necessarily require hospitalization. Second, we could not take into account the women’s medical history before 2007, as French hospital data were unable to identify in a reliable manner events prior to 2007.

The strength of this study lies in the ability of the French hospital database to capture important maternal information for all deliveries in France. In previous studies, Quantin et al. compared such information from various sources and showed that it was possible to identify deliveries in mainland France with a difference of 0.3 % compared with the civil registry, which records all births in our country [33]. In a pilot study in 2012 based on 20 cases of GDM from 300 medical records [34], Pierron et al. also assessed the ability of French hospital data to identify GDM and found a positive predictive value of 88.9 % [74.3–100].

French hospital data allowed the satisfactory, though not optimal, identification of women who had GDM. Our results showed a prevalence of 4.14 %, which is in keeping with the prevalence between 2 and 6 % in France and Europe [2].

Our results regarding the hypertensive risk associated with a history of GDM were similar to those of previous studies [16–35]. A retrospective study carried out between 1998 and 2007 in Massachusetts found an increased risk of hypertensive disease in women with a history of GDM after adjustment for several CVD risk factors (HR 1.75 [1.28–2.37]) [35]. Another study reported an 88 % increased risk of hypertension in such women after adjusting for age, menopausal status, and race/ethnicity (OR 1.88 [1.34–2.64]) [16].

In agreement with our results, a scoping review [17] which included studies by Carr et al. [16.] and Freibert et al. [36], concluded that the association between history of GDM and stroke was inconclusive.

As regards cardiovascular diseases, most studies showed an overall increase in CVD risk beyond 10 years postpartum [15, 16, 37]. In a case–control study, the adjusted odds ratio for the association of GDM with cardiovascular disease was 1.51 [1.07–2.14] for a mean follow-up of 9.1 years. [37]. In a population-based cohort study, Shah et al. found that CVD risk increased in the 11.5 years following the index pregnancy (HR 1.71 [1.08–2.69]) but the association was attenuated when adjusted for subsequent diabetes (HR 1.13 [0.67–1.89]) [15]. Only one study concluded that GDM increased self-reported CVD in young women (45.5 years on average) who had first-degree relatives with T2DM and this independently of T2DM in the women themselves (aOR = 1.85 [1.21–2.82]) [16].

To our knowledge, no study has shown that women with a history of GDM had a higher risk of being hospitalized for CVD events during the 7 years following delivery, regardless of subsequent DM, obesity and hypertensive disorders of pregnancy. Our results showed that the early increase in CVD risk cannot be exclusively attributable to the subsequent development of diabetes. Although our study included only hospitalized women, who can be considered less healthy, these findings are consistent with previous studies that estimated cardiovascular risk in the short term after pregnancy [18, 19, 22]. One study showed an increased risk of cardiac dysfunction in women with GDM at least 1 year after delivery [22]. Similarly, according to Gunderson et al., a history of GDM can be considered a risk factor for atherosclerosis before the onset of diabetes or metabolic syndrome [18].

The prevalence of CVD in women with GDM remains low up to 7 years after delivery. Only 90 of the 62,958 women with a history of GDM experienced coronary vascular diseases. This small number of individuals cannot justify systematic screening for coronary heart disease or the inclusion of GDM history in the risk profile.

However, the increased risk of CVD does lead us to recommend the control of other risk factors and lifestyle changes from postpartum onwards. As pregnancy is a period during which women are particularly aware of own health behavior and that of their children, it offers a window of opportunity for clinicians to act and propose lifestyle changes, for example. A partnership between clinicians and women with a history of GDM should be encouraged. This period of safe behavior may be extended by appropriate support programs which take into account the living conditions of working women with young children [38, 39]. The central idea is to engage women in a health education approach to promote healthy ageing.

In conclusion, our study showed for the first time that GDM is an independent factor risk of CVD in the 7 years following delivery. It suggests that effective management of risk factors should be implemented in these women from postpartum onwards.
